# A ribonuclease activity linked to DYW1 in vitro is inhibited by RIP/MORF proteins

**DOI:** 10.1038/s41598-023-36969-6

**Published:** 2023-07-03

**Authors:** Robert D. Boyd, Michael L. Hayes

**Affiliations:** 1 Department of Chemistry, University of Illinois at Urbana–Champaign, Urbana, IL 61801 USA; 2 Department of Chemistry and Biochemistry, California State University Los Angeles, Los Angeles, CA 90032 USA

**Keywords:** Biochemistry, Enzymes, Hydrolases, RNA decay, RNA editing

## Abstract

Organellar C-to-U RNA editing in plants occurs in complexes composed of various classes of nuclear-encoded proteins. DYW-deaminases are zinc metalloenzymes that catalyze hydrolytic deamination required for C-to-U modification editing. Solved crystal structures for DYW-deaminase domains display all structural features consistent with a canonical cytidine deamination mechanism. However, some recombinant DYW-deaminases from plants have been associated with ribonuclease activity in vitro. Direct ribonuclease activity by an editing factor is confounding since it is not required for deamination of cytosine, theoretically would be inimical for mRNA editing, and does not have a clear physiological function in vivo. His-tagged recombinant DYW1 from *Arabidopsis thaliana* (r*At*DYW1) was expressed and purified using immobilized metal affinity chromatography (IMAC). Fluorescently labeled RNA oligonucleotides were incubated with recombinant *At*DYW1 under different conditions. Percent relative cleavage of RNA probes was recorded at multiple time points from triplicate reactions. The effects of treatment with zinc chelators EDTA and 1, 10-phenanthroline were examined for r*At*DYW1. Recombinant His-tagged RNA editing factors *At*RIP2, *Zm*RIP9, *At*RIP9, *At*OZ1, *At*CRR4, and *At*ORRM1 were expressed in *E. coli* and purified. Ribonuclease activity was assayed for r*At*DYW1 in the presence of different editing factors. Lastly, the effects on nuclease activity in the presence of nucleotides and modified nucleosides were investigated. RNA cleavage observed in this study was linked to the recombinant editing factor r*At*DYW1 in vitro. The cleavage reaction is sensitive to high concentrations of zinc chelators, indicating a role for zinc ions for activity. The addition of equal molar concentrations of recombinant RIP/MORF proteins reduced cleavage activity associated with r*At*DYW1. However, addition of equal molar concentrations of purified recombinant editing complex proteins *At*CRR4, *At*ORRM1, and *At*OZ1 did not strongly inhibit ribonuclease activity on RNAs lacking an *At*CRR4 cis-element. Though *At*CRR4 inhibited *At*DYW1 activity for oligonucleotides with a cognate cis-element. The observation that editing factors limit ribonuclease activity of r*At*DYW1 in vitro*,* suggests that nuclease activities are limited to RNAs in absence of native editing complex partners. Purified r*At*DYW1 was associated with the hydrolysis of RNA in vitro*,* and activity was specifically inhibited by RNA editing factors.

## Introduction

There are hundreds of genes encoding serial pentatricopeptide repeats (PPRs) in a typical higher plant and this family of genes is essential for various steps of RNA maturation^1,2^. Many PPR genes in plants also encode a C-terminal domain dubbed the DYW-deaminase domain (Pfam: Pf14432) after the common terminal tripeptide sequence motif aspartate-tyrosine-tryptophan (DYW) and this domain along with upstream regions share common features with characterized nucleotide deaminases^3–5^. The DYW-deaminase domain coordinates two zinc ions per DYW-deaminase subunit^4,6^ through a conserved [(H/C)xEx_(25–30)_CxxC] motif common to other nucleotide deaminases and an unique [Hx_22_Hx_6_CSC] zinc binding domain. Genetic complementation studies indicate the necessity of zinc binding domains and the proposed catalytic glutamate for RNA editing^7–9^. Cementing the role in editing, expression of moss *Physcomitrella patens* PPR proteins PPR65 and PPR56 are sufficient for specifically editing their RNA targets in an *Escherichia coli* expression system^10^ and purified recombinant PPR65 protein also can edit RNAs in vitro^11^.

In addition to the clear role for the DYW-deaminase in RNA editing, additional roles in transcript maturation have been proposed. The PPR protein with a C-terminal DYW-deaminase domain called CHLORORESPIRATORY REDUCTION2 (CRR2) is required for the accumulation of processed *rps7* and *ndhB* mRNAs^12^. Failure of native mRNA termini to accumulate in *crr2* knock-out plants results in the inactivity of the NDH complex^12^. Investigation of RNA sequences in wildtype plants compared to knock-out plants has failed to reveal a direct role in RNA editing for CRR2^13^. However, ribonuclease activity observed in vitro for a recombinant protein with the DYW-deaminase domain of CRR2 was interpreted as evidence for direct endoribonuclease activity in the *rps7*-*ndhB* intergenic region^14^. Complicating a simple direct role in sequence specific cleavage, PPR tracts, like many RNA binding domains, protect RNAs from ribonucleases allowing for the observation of “protected” footprints^15^. Thus, it can be difficult to discriminate if specific transcripts result from protection from a nuclease acting in trans or from a direct sequence specific cleavage nearby a cis-element. The observation of CRR2 dependent “footprints” at both the 5′ end of *ndhB* and 3′ end of *rps7* transcripts are strongly suggestive of a protective role from nucleases by the CRR2 PPR tract^13^. Thus, a direct physiologic role for the DYW-deaminase domain associated ribonuclease cleavage remains controversial.

Recently the crystal structure for *At*DYW1 has been solved^16^ and two additional solved crystal structures for the DYW domain of editing factor *At*OTP86 have been reported: a tetrameric structure called “active” (PDB 10.2210/pdb7O4F/pdb) and a dimeric structure called “inactive” (PDB 10.2210/pdb7O4E/pdb)^6^. The “active” conformation has an active site with features consistent with other cytidine deaminases, however the “inactive” conformation likely cannot accommodate a cytidine base substrate due to steric occlusion^6^. Addition of tetrahydrouridine (THU) during the preparation of a recombinant DYW-deaminase protein was critical for the observation of the “active” conformation but the molecule was not observed in the solved structure^6^. In addition to THU, the nucleotide ATP was also linked to the “active” conformation^6^. Additionally, the crystal structure of *At*DYW1 suggests interactions with the PPR *At*CRR4 form the substrate binding site^16^. A nuclease-like domain has not been described for the DYW-deaminase in either conformation.

At least three recombinant DYW domain-containing proteins have been linked to ribonuclease activity in vitro: the aforementioned CRR2^14^; At2g02980^17^ which was later recognized to be a chloroplast RNA editing factor named Organelle Transcript Processing 85 (OTP85)^18^; and Os05g30710^17^. The two reports of DYW-associated ribonuclease activity indicate sensitivity to extremely high concentrations (100–200 mM) of the metal chelator EDTA^17^. Cleavages linked to the DYW domain of *At*CRR2 precede adenines (As) in absence of the native PPR tract, which demonstrates some general sequence affinity by the domain alone. Sequence specificity for local bases nearby the targeted cytidine for the RNA editing apparatus has been well documented^19–21^ and recently more precisely shown to relate to the DYW domain^22^. It is unclear what residues of the DYW domain might be responsible for nuclease activity and the metastable nature of RNA and the simplicity of catalysis yields many possibilities.

In their native environment, PPR proteins with DYW-deaminase domains (PPR-DYWs) have been physically linked to several other proteins that are necessary for RNA editing in *Arabidopsis thaliana*^23^ and *Zea mays*^24^. RNA editing likely occurs in large heterocomplexes that include PPR proteins, DYW-deaminase domains, RNA editing factor interacting proteins/multiple organellar RNA editing factors RIP/MORFs, organellar zinc-finger (OZ) proteins, and organelle RNA recognition motif (ORRM) proteins^23^. RIP/MORFs bind PPR proteins^25,26^ extensively through L-motif interactions of P-L-S motifs^27^ and have been shown to interact with the E and E+ motifs^28^ present in the broadly defined enzymatic domain^3^ that includes the DYW-deaminase domain.

In this study, recombinant *At*DYW1 was expressed and purified to investigate nuclease activity associated with an RNA editing enzyme in vitro. The editing factor *At*DYW1 is essential for creating the start codon of *ndhD* transcripts in *Arabidopsis*^29^. A ribonuclease activity assay was constructed using Cy-5 labeled oligoribonucleotides with various sequences. In absence of conflict with the established role of *At*DYW1 in editing *ndhD* transcripts in vivo^29^, RNA cleavage of *ndhD* sequence containing oligonucleotides in vitro was specifically reduced in the presence of *At*CRR4. In oligonucleotides without *At*CRR4 cis-elements only RIP family editing complex members could inhibit r*At*DYW1 nuclease activity. RIP proteins could not protect RNAs from cleavage catalyzed by RNaseA. Zinc chelators strongly inhibit ribonuclease activity. Nucleotides and modified nucleosides that favor formation of “active” conformations of the DYW-deaminase did not alter ribonuclease activity and editing activity could not be reconstituted in vitro. Therefore, the zinc-dependent nuclease activity associated with the DYW-deaminase is greatly reduced in the context of editing complex member association.

## Results

The editing factor DYW1 from *Arabidopsis thaliana* was examined due to a few unique features that make it amenable for this study versus other DYW-deaminase containing proteins. *At*DYW1 does not possess an N-terminal PPR-tract, only a degenerate PPR-like N-terminal region as another editing factor *At*CRR4 is required to specifically edit *ndhD* transcripts in vivo^29^. Since the *At*DYW1 does not have a lengthy PPR-tract, the protein theoretically would not have a strong sequence preference to protect a region of an RNA probe through PPR shielding. All other DYW-deaminase domain containing editing factors in *Arabidopsis* have N-terminal PPRs. Also, *At*DYW1 could be studied as an entire protein not as a C-terminal protein fragment with potentially altered solvent exposure that might affect its tertiary structure. *At*DYW1 is not known to be associated with RNA cleavage around the singular *ndhD* editing target suggesting no coupling between editing and ribonuclease cleavage in vivo.

**Figure 1:**
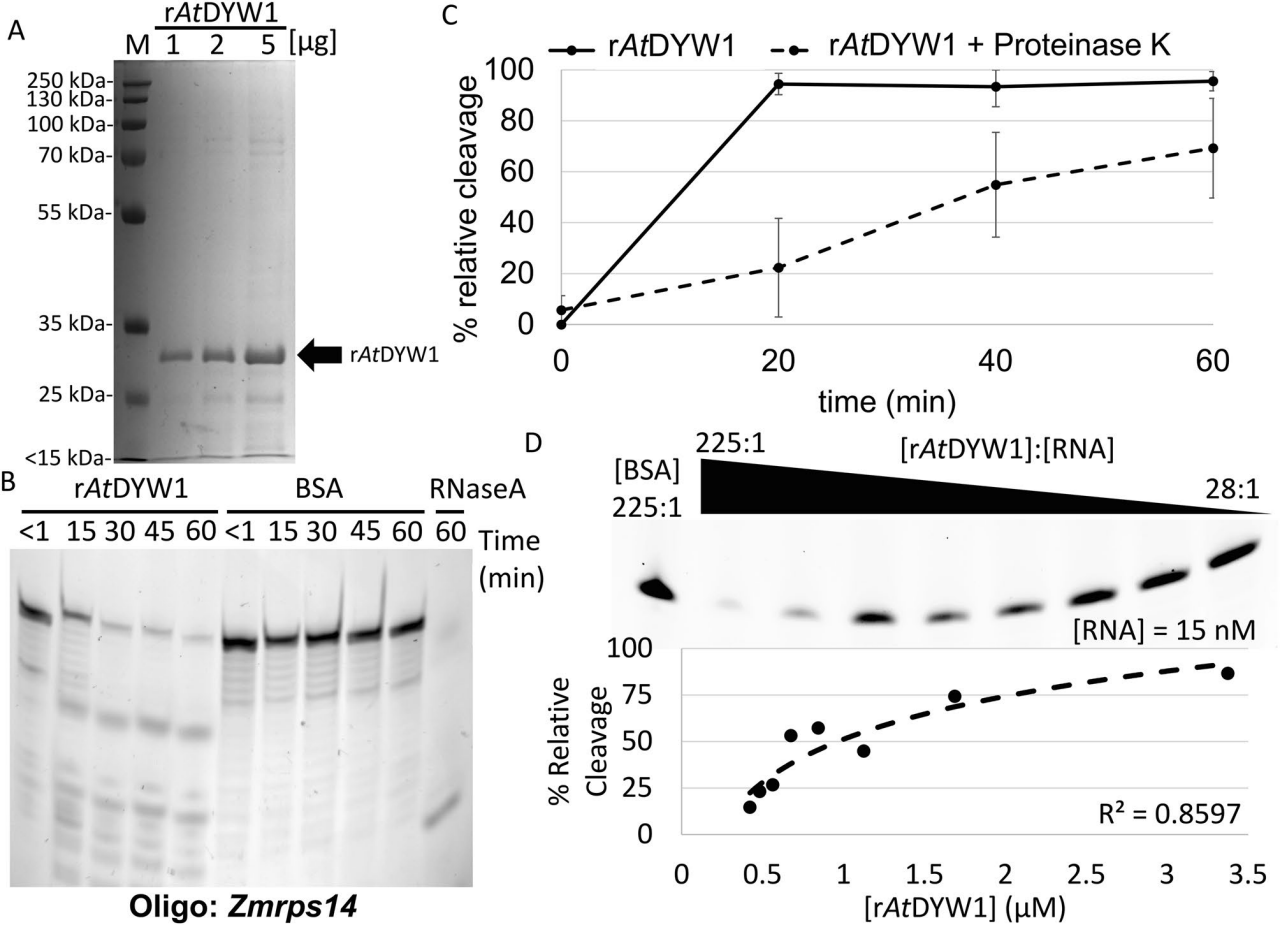
Recombinant *At*DYW1 has nuclease activity in vitro. (**A**) An image of a Coomassie-stained SDS-PAGE to assess purity of the IMAC purified r*At*DYW1 with 1, 2, and 5 μg loaded per lane. (**B**) RNA oligonucleotides labeled with a 5′ Cy-5 fluorophore visualized after separation on a 6 M Urea 20% Polyacrylamide gel from assay of nuclease activity. From left to right lanes represent rAtDYW1 and BSA were incubated from < 1 to 60 min followed by a 2.5 μg/mL RNaseA control at 60 min. (**C**) Relative cleavage $\left[\frac{(\text{Intensity at }0\text{ min }-\text{ Intesity at timepoint})}{\text{Intensity at }0\text{ min}} \times 100\right]$ was calculated and plotted in an X-ray scatterplot for timepoints 20, 40, and 60 min for r*At*DYW1 and an equivalent reaction with addition of 40 U/mL of Proteinase K. Error bars represent 1 standard deviation of the mean for three separate reaction replicates. (**D**) Ribonuclease activity was assayed in reactions with a titration of r*At*DYW1 from 225 to 28:1 molar ratio compared to labeled RNA probe at 15 nM. Dotted trendline is a best fit line using a logarithmic fit. For (**B**–**D**) the oligo *Zmrps14* was used.

Recombinant *At*DYW1 was expressed in *E. coli* with a N-terminal 6X-His tag (r*At*DYW1) to determine if ribonuclease activity is a common feature for the DYW-deaminase domain. Ample amounts of the protein could be obtained with high purity from a single round of immobilized metal affinity chromatography (Fig. 1A, Fig. S1). Purified proteins resolved by gel filtration combined with a multiple angle light scattering estimated a molecular weight consistent with a monomer (Fig. S2).

Recombinant *At*DYW1 fractions were initially assayed for ribonuclease activity on a 5′ Cy-5 labeled 26-mer RNA oligonucleotide called Zmrps14 with the sequence (5′-Cy-5-UCAUUUGAUUCGUCGAUCCUCAAAAA) derived from the *rps14* gene present in the chloroplast genome of *Zea mays*. The RNA oligo was chosen since it would allow the study of ribonuclease activity on a general RNA sequence. Ribonuclease activity was observed relative to a bovine serum albumin (BSA) negative control with a distinct pattern of cleavage products compared to RNaseA (Fig. 1B). Addition of 1 U/μL Ribolock ribonuclease inhibitor was found to improve the consistency of the assay and was maintained throughout all reactions. Ribonucleases are ubiquitous in the environment and Ribolock was sufficient to reduce background cleavage. Proteinase K was added to 40 U/mL in reactions to investigate if cleavage was due to protein and not free ions, and a general decline in percent relative cleavage was observed (Fig. 1C). Recombinant *At*DYW1 was titrated in a series of reactions with molar ratios of r*At*DYW1 versus RNA from 225:1 to 28:1 and a logarithmic trend was observed with greater nuclease activity correlating with increased r*At*DYW1 concentration (Fig. 1D).

**Figure 2:**
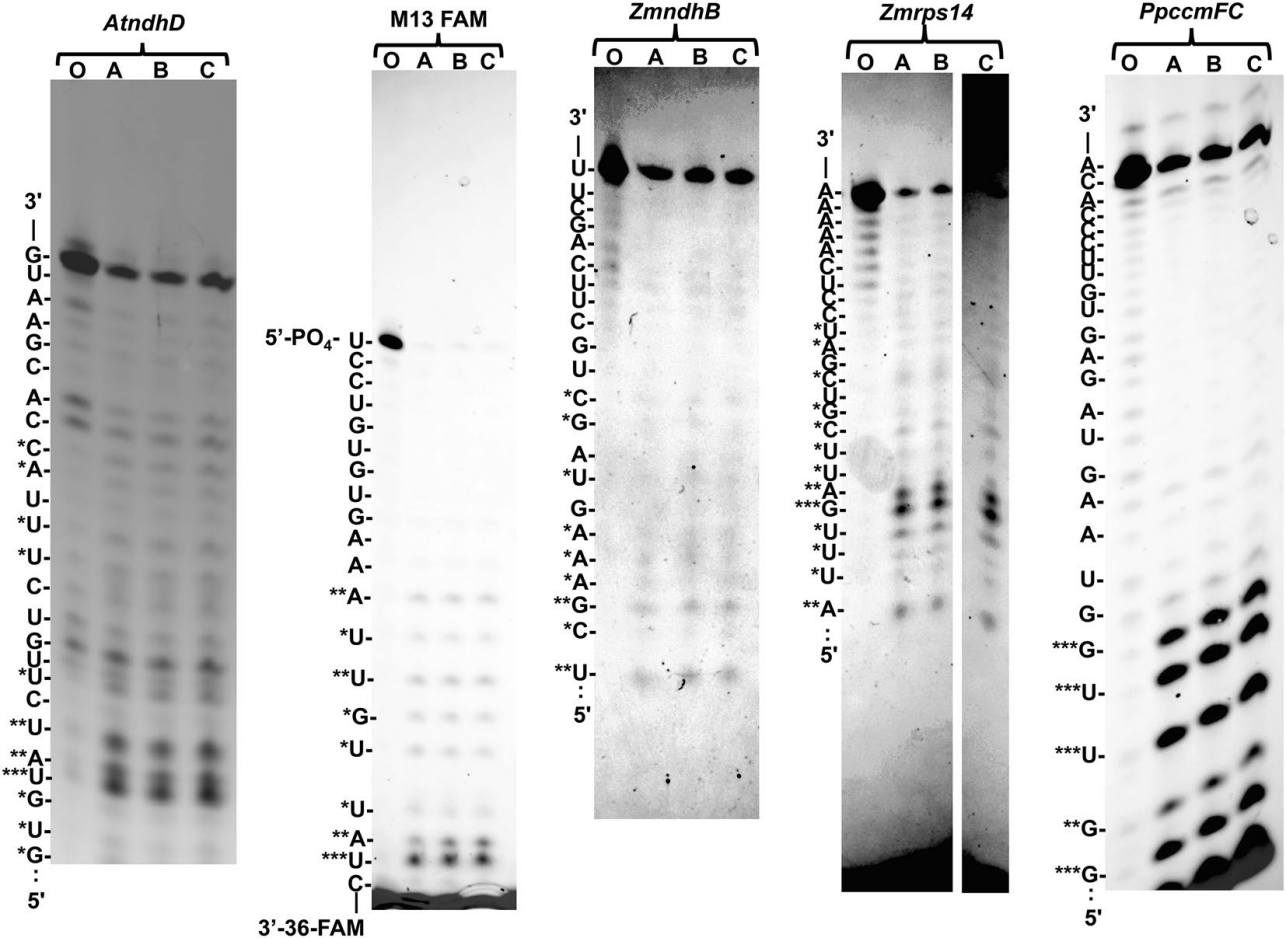
*At*DYW1 was incubated with 5 different RNA oligonucleotides (*AtndhD*, M13 FAM, *ZmndhB*, *Zmrps14*, and *PpccmFC*) at 28 °C for 20 min in triplicate reactions (lanes A–C) and compared to the oligonucleotide without incubation with DYW1 (lanes O). RNA species from reactions were separated on a 20% polyacrylamide gel in 6 M urea and imaged using an Azure c400 imager using channels for each specific fluorophore. At left of each gel image, letters represent the likely 3′ nucleotide identify. Bands were ranked as highly (***), moderately (**), and slightly (*) increased species compared to the oligonucleotide control.

Oligonucleotides with different sequences were incubated with r*At*DYW1 to test specificity of nuclease activity through the creation of new RNA species: (1) A 5′ Cy-5 labeled *AtndhD* (5′-Cy-5-GGUGUAUCUUGUCUUUACCACGAAUG-3′) oligonucleotide has the cis-element for *At*CRR4 with a sequence context of 20 nucleotides upstream and 5 nucleotides downstream of the editing site; (2) an oligonucleotide M13 FAM (5′-UCCUGUGUGAAAUUGUUAUC-FAM-3′) with a 3′ fluorescein moiety; (3) A 5′ Cy-5 labelled *ZmndhB* (5′-Cy-5-UACUUCGAAAGUAGCUGCUUCAGCUU-3′) oligonucleotide with sequence around another editing site in maize; (4) the aforementioned *Zmrps14* oligonucleotide; and (5) A 5′ Tetrachlorofluorescein labelled *PpccmFC* (5′-TET-UGGUUGGUAAGUAGAGAUGUUCCCACA-3′) oligonucleotide with sequence around an editing site in *Physcomitrella patens* mitochondria. The patterns of generated RNA species after incubation with r*At*DYW1 were distinct between the 5 oligonucleotides (Fig. 2, Fig. S3). The projected positions of cleavage did not demonstrate a robust sequence preference but a more general hydrolysis resulting in 8-mers or shorter RNA species.

**Figure 3:**
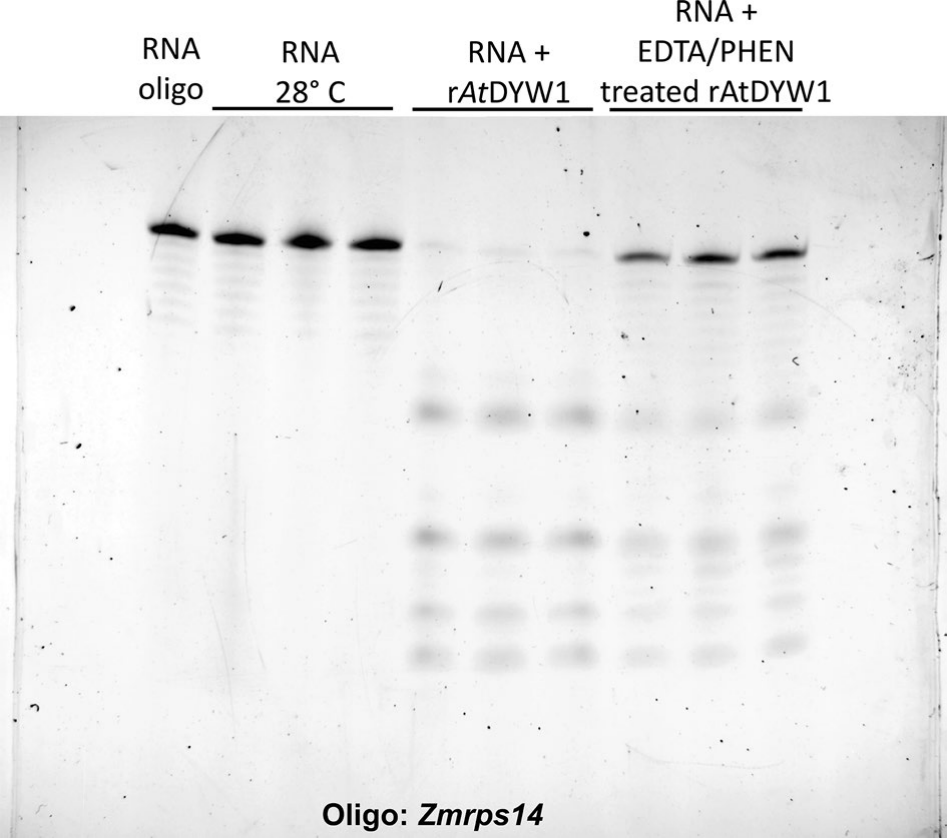
Ribonuclease activity for r*At*DYW1 is sensitive to treatment with zinc ion chelators. Fractions of r*At*DYW1 were dialyzed for 3 days with and without an inhibitor cocktail of 25 mM EDTA and 0.65 mM 1,10-phenanthroline. Ribonuclease activity was assayed for dialyzed r*At*DYW1 fractions. At top, a picture of RNA oligos with the *Zmrps14* sequence separated on a 6 M urea 20% PAGE. Lanes from left to right represent an RNA oligo control, triplicate reactions of the RNA oligo subjected to reaction conditions without enzyme, RNA oligonucleotides treated with r*At*DYW1 dialyzed 3 days at 4 °C without chelator, and RNA treated with r*At*DYW1 dialyzed in a buffer containing EDTA and 1,10-phenanthroline.

Prior experiments examining ribonuclease activity of the DYW-deaminase domain suggested a role for metal ions in catalysis based on titration of the metal chelator EDTA^14,17^. Recombinant *At*DYW1 is known to bind two zinc ions per polypeptide chain^4^. Fractions of purified r*At*DYW1 were dialyzed in a buffer with and without divalent metal chelators (25 mM EDTA, 650 μM 1,10-phenanthroline) for three days. Ribonuclease activity was lower in fractions treated with metal chelators versus without, but activity could not be completely eliminated (Fig. 3).

Several editing factors were cloned and expressed to investigate their effect on r*At*DYW1 linked activities. Editing factors were chosen based on a possible editosome responsible for the creation of the initiation codon of the chloroplast gene *ndhD*: (1) the PPR *At*CRR4 likely binds to the cis-element upstream of the aCg > aUg editing target; (2) *At*DYW1 acts as a deaminase enzyme; (3) RIP2 and RIP9 are theorized to bind L-domains of the PPR or E-domains nearby the DYW domain; and (4) *At*OZ1 and *At*ORRM1 act as critical accessory factors.

**Figure 4:**
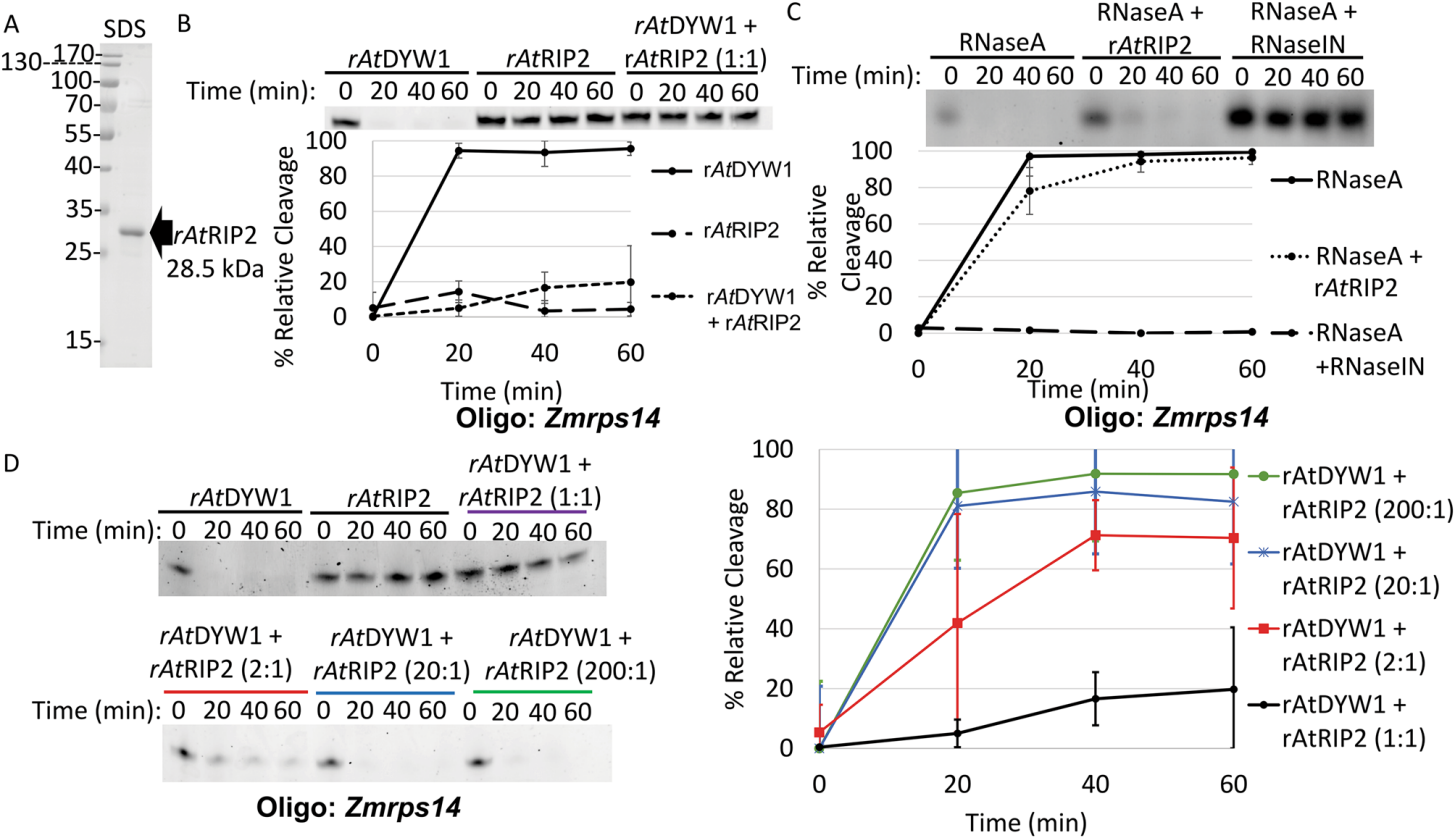
Recombinant *At*RIP2 inhibits ribonuclease activity of r*At*DYW1. (**A**) The Coomassie stained SDS-PAGE image indicates the purity of r*At*RIP2 fractions after one round of Ni-NTA affinity chromatography and one round of size exclusion chromatography. (**B**) Ribonuclease activity on RNA oligonucleotides was assayed at 0 min, 20 min, 40 min and 60 min in triplicate reactions (representative image from one time course shown) in the presence of r*At*DYW1, r*At*RIP2, and r*At*DYW1 mixed with r*At*RIP2 in an equimolar ratio (1:1). At top a representative image is shown of the set of the 6 M urea 20% PAGE used to quantify percent relative cleavage. Below a graph displays % relative cleavage of all triplicate reactions across 4 timepoints. (**C**) Ribonuclease activity was assayed for triplicate reactions containing 2.5 μg/mL RNaseA, 2.5 ng/μL RNaseA + 5 μM r*At*RIP2, and 2.5 μg/mL RNaseA + 4 U/μL Ribonuclease inhibitor (RNaseIN). At top a representative image of one time-course reaction is shown and below a X–Y scatterplot displays % relative cleavage as a function of time for triplicate reactions. (**D**) Activity was measured in triplicate reactions with various stoichiometric ratios of rAtRIP2 versus r*At*DYW1 from (1:1 to 1:200). Representative gel images are shown at left and % relative cleavage for triplicate reactions are represented in a X–Y scatterplot at right. (**B–D**) Error bars represent 1 standard deviation from the mean for triplicate reaction replicates. (**B**–**D**) used the oligo *Zmrps14*.

Recombinant *At*RIP2 could be purified in large quantities (Fig. 4A, Fig. S4). Ribonuclease activity was absent when r*At*RIP2 was added alone in equivalent molar amounts as r*At*DYW1. However, when equimolar amounts of both r*At*DYW1 and r*At*RIP2 were added together, little hydrolysis of RNA oligonucleotides was observed (Fig. 4B). The reduction in nuclease activity by r*At*RIP2 could either be due to direct protection of RNA or a specific interaction with r*At*DYW1. RNA hydrolysis was robust after the addition of 2.5 ng/μL of RNaseA to reactions (Fig. 4C). RNaseA catalyzed nuclease activity remained robust after the addition of 5 μM r*At*RIP2, however little hydrolysis was observed for equivalent reactions containing 4 U/μL Ribolock RNase Inhibitor (Fig. 4C). The inhibitory effect of r*At*RIP2 on nuclease activity was reduced in reactions with molar excess of r*At*DYW1 and a 1:1 molar ratio elicited the greatest protection (Fig. 4D).

**Figure 5:**
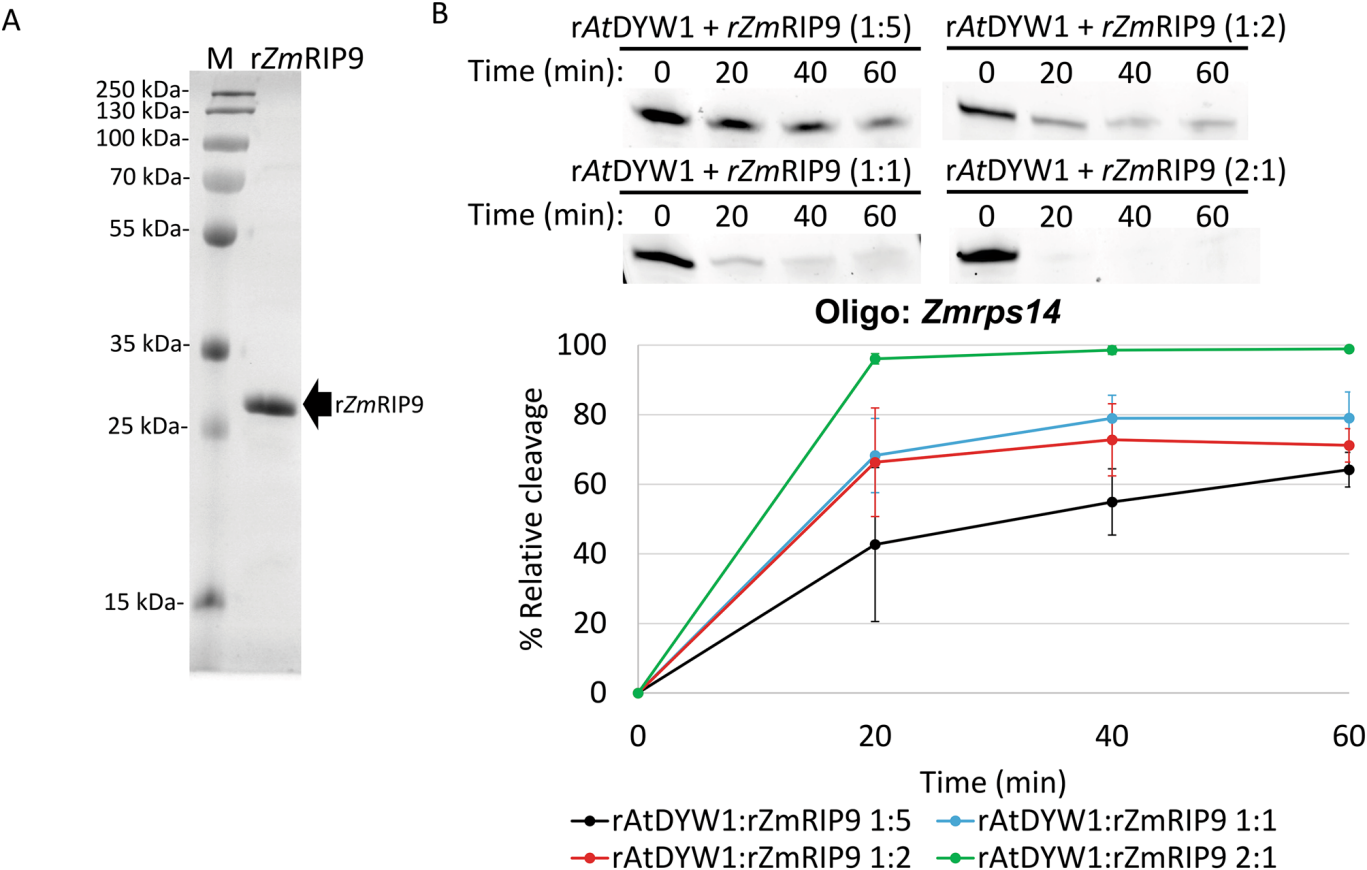
Recombinant *Zm*RIP9 progressively inhibits ribonuclease activity of r*At*DYW1 at increasing relative molar ratios. (**A**) Recombinant *Zm*RIP9 was purified using Ni-NTA affinity followed by size exclusion chromatography steps. An image of a representative Coomassie SDS-PAGE indicates purity. (**B**) At top, ribonuclease activity was assayed for triplicate reactions at 0, 20, 40, and 60 min timepoints containing r*Zm*RIP9 mixed with r*At*DYW1 with stoichiometric ratios of 5:1, 2:1, 1:1, and 1:2. Below, a X–Y scatterplot displays % relative cleavage at 4 timepoints. Error bars represent 1 standard deviation from the mean for triplicate reactions. (**B**) Nuclease reaction use the *Zmrps14* oligo.

Several attempts at expression and purification of the RIP/MORF family member r*At*RIP9 led to small protein yields with many degraded proteins present in enriched fractions (Fig. S5). Addition of approximately equimolar quantities of r*At*RIP9 to r*At*DYW1 led to reduced hydrolysis of RNA oligonucleotides compared to equivalent reactions with only r*At*DYW1 (Fig. S5b). Expression and purification of the *Zea mays* ortholog r*Zm*RIP9 yielded large quantities of stable protein (Fig. 5A, Fig. S6). When r*Zm*RIP9 was added to nuclease reactions in increasing molar ratios with a static concentration of r*At*DYW1 decreasing amounts of relative cleavage was observed (Fig. 5B).

**Figure 6:**
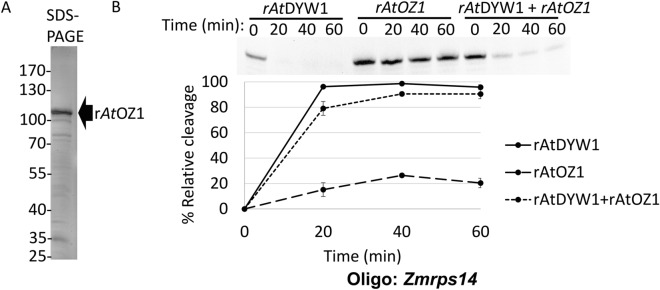
The editing factor r*At*OZ1 does not strongly inhibit r*At*DYW1 at an equimolar ratio. (**A**) Recombinant *At*OZ1 was purified using a single round of IMAC and an SDS-PAGE image is shown to evaluate protein purity. (**B**) Ribonuclease activity was assayed for triplicate reactions containing r*At*DYW1, r*At*OZ1, and rAtDYW1 + rAtOZ1 mixed in an equimolar ratio on the oligonucleotide *Zmrps14*. A representative image of a 6 M urea 20% PAGE is shown at top and the data from the triplicate reactions is represented in the X–Y scatterplot below. (**B**) Error bars represent 1 standard deviation from the mean.

Other editing factors were expressed and purified to examine their effects on r*At*DYW1 linked RNA hydrolysis. The zinc finger protein r*At*OZ1 could be purified (Fig. 6A, Fig. S7) and despite some hydrolysis in the absence of r*At*DYW1, extensive hydrolysis was observed in reactions with equimolar concentrations with r*At*DYW1 (Fig. 6B). Recombinant r*At*ORRM1 could be purified in low amounts in the presence of degradation products (Fig. S7). There was no difference in nuclease activity of reactions with around equimolar amounts of r*At*ORRM1 and r*At*DYW1 versus r*At*DYW1 alone (Fig. S7c).

**Figure 7:**
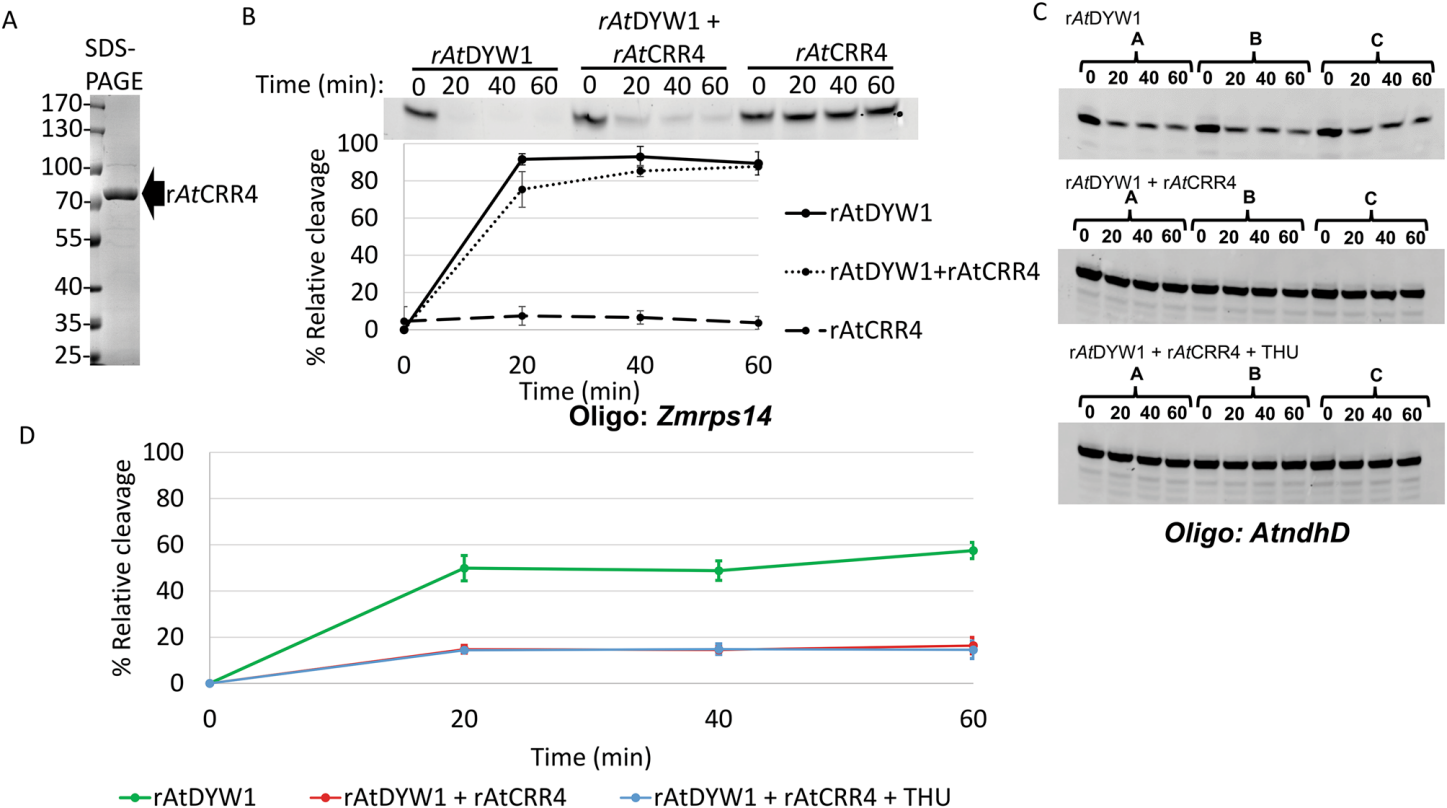
Recombinant *At*DYW1 ribonuclease activity was reduced on oligonucleotides with *AtndhD* sequences when incubated with r*At*CRR4 but not affected by not affected by addition of nucleotides or tetrahydrouridine. (**A**) Recombinant *At*CRR4 was purified by IMAC followed by gel filtration and the SDS-PAGE image relates purity. (**B**) Hydrolysis of RNA *Zmrps14* oligonucleotides was assayed in triplicate reactions with r*At*DYW1, r*At*DYW1 + r*At*CRR4 mixed in an equimolar ratio, and r*At*CRR4. At top a representative image of RNA oligonucleotides separated on a 6 M urea 20% PAGE. Below a scatterplot represents % relative cleavage at 4 time points for the triplicate reactions. (**C**) Images of 20% PAGE in 6 M urea show RNA species created through out a 0, 20, 40, 60 min time course from incubation of *AtndhD* oligonucleotides with r*At*DYW1 alone (top), r*At*DYW1 with equimolar r*At*CRR4 (middle), and r*At*DYW1 with equimolar r*At*CRR4 in the presence of THU. Reactions were run in triplicate and lanes are labeled for each reaction with (**A–C**). (**D**) % relative cleavage was plotted versus time calculated from the intensity of bands from the gel using ImageJ. Error bars represent one standard deviation from the mean for triplicate reactions. (**B,D**) Error bars represent 1 standard deviation from the mean.

The binding partner with *At*DYW1 called *At*CRR4 was expressed and purified (Fig. 7A; Fig. S7). The PPR protein *At*CRR4 does not possess a DYW-deaminase domain and acts as a specificity factor for *At*DYW1 in an editing complex on *AtndhD* transcripts. Hydrolysis of the *Zmrps14* RNA probe lacking the *At*CRR4 cis-element was minimal after 60 min in the presence of recombinant *At*CRR4 (Fig. 7B). Percent relative cleavage of RNA in reactions with equimolar amounts of r*At*CRR4 and r*At*DYW1 were comparable to reactions with r*At*DYW1 alone (Fig. 7B).

An oligonucleotide with the sequence containing the cis-element for *At*CRR4 and the native editing target for *At*DYW1 was assayed for hydrolysis by r*At*DYW1. Hydrolysis was observed in the presence of r*At*DYW1 but was reduced in the presence of r*At*CRR4 (Fig. 7C,D). The pattern of resulting RNA species produced by cleavage changed only slightly with the addition of r*At*CRR4, which resulted in increased stability of full-length oligonucleotides and RNAs ending in A24-G22 (Fig. S9). Based on the crystal structure of the *At*DYW1 it has been hypothesized that a *At*DYW1–*At*CRR4 complex creates the substrate binding site^16^. The addition of 2 mM THU promoted the “active” substrate binding site for the DYW domain from OTP86^6^. Addition of 2 mM THU to nuclease reactions containing both r*At*DYW1 and r*At*CRR4 did not affect the rate of cleavage nor the resulting RNA species profile (Fig. S9). Additionally, RNA hydrolysis was equivalent in reactions with r*At*DYW1 alone under standard conditions with 2 mM THU, ATP, and CMP (Fig. S10a).

Since most of the components of the theoretical editing complex were at hand, an attempt was made to reconstruct an active editosome in vitro by combining all purified editing factors. Unfortunately, conversion of an RNA with *ndhD* sequences was not observed in vitro (Fig. S10b).

## Discussion

### DYW-deaminases domains are most likely not exclusively either a C-to-U editase or a ribonuclease enzyme

It has been posited that the DYW-deaminase domain might be adapted to either RNA editing or RNA cleavage functions^14^. This was largely based on initial findings that the PPR protein with the DYW-deaminase domain called *At*CRR2 had been found to be necessary for transcript maturation without any known editing function, and at the time, no editing factors were known to be directly linked to ribonuclease activity^14,17^. Conflicting with this hypothesis, one PPR-DYW protein found to have ribonuclease activity At2g02980^17^ was later named OTP85 and found necessary for an RNA editing site in *ndhD* transcripts^18^. Our results add additional support against the hypothesis of distinct molecular functions for the DYW-deaminase as *At*DYW1 is clearly an editing factor^29^ with ribonuclease activity in vitro (Fig. 1, Fig. S1). There is no conflict with the C-to-U editing enzyme function in vivo based on the cleavage activity observed in vitro as the contexts are fundamentally different. Native editing complexes are known to contain many different proteins in unknown stoichiometries. Despite the potentially artifactual nature of the ribonuclease activity observed for the DYW-deaminase in vitro, ribonuclease activity has been observed for at least 4 recombinant DYW-deaminase proteins. Specific features of the domain likely lead to these observations as equivalently expressed and purified proteins did not possess a similar activity.

### The DYW-deaminase as a bifunctional enzymatic domain?

It is attractive to posit that based on evidence of nuclease activity in vitro and confirmed editing activity in vivo that *At*DYW1 is a bifunctional enzyme. However, in this study, equimolar concentrations of *At*DYW1 with editing factors of the RIP family almost completely abrogate nuclease activity. Also, if the RNA substrate has a cis-element for *At*CRR4 than nuclease activity is reduced in the presence of r*At*CRR4. Since RIP family proteins have been found to tightly bind PPR proteins^23^, probably through PPR L-motifs^27^ and E/E+ motifs^28^, it is likely that in organelles PPR-DYW proteins are commonly associated with RIP proteins. Additional evidence is required to directly link the DYW-deaminase domain with ribonuclease cleavage in vivo.

As improved structural information becomes available for the domain, perhaps the biochemical mechanism responsible for nuclease activity might become apparent. The solved crystal structures for the DYW-deaminase domain indicate “active” and “inactive” conformations^6^. The active conformation has all the features consistent with a nucleotide deaminase enzyme confirming the results from several biochemical studies^7–11^. No function has been ascribed to the “inactive” conformation. Equipped with a model of the DYW-deaminase bound to an RNA ligand, it might be possible to identify possible interactions associated with nuclease activity.

This study did not investigate the physiological function of the ribonuclease activity linked to *At*DYW1. It is unlikely to relate to editing of *ndhD* transcripts. The nuclease activity does not require *ndhD* sequences or PPR specificity factors. There is some controversy over the physiological function of the DYW-deaminase domain of *At*CRR2 where RNA cleavage dependent on the PPR-DYW was first postulated^12^. Subsequently, ribonuclease activity was demonstrated by the DYW-deaminase domain^17^. A recent study that focused on transcripts bound to *At*CRR2 was dismissive of direct nuclease activity in vivo largely due to the observation that both native 5’ and 3’ transcript termini contain the *At*CRR2 cis-element^13^. Since RIP/MORF proteins reduce cleavage of at least one DYW-deaminase in vitro, the discrepancy between absence of specific cleavage observed in vivo and general cleavage in vitro for *At*CRR2 might be explained by the relative presence in vivo or absence in vitro of the PPR tract and RIP/MORF proteins.

### Speculative potential roles of DYW-deaminase linked nuclease activity

Though our findings are not able to indicate a specific biological function for DYW-deaminase linked ribonuclease activity, the activity has been observed in vitro. This could be due to an artifact in nonnative conditions or a hidden biological function. Perhaps DYW-domains estranged from editing complexes might participate in RNA degradation instead of deamination. Degradation of RNAs outside of the editosome might confer some additional protection from unwanted off-target C-to-U changes due to promiscuous deaminase activity. This speculative biological activity might be difficult to separate from general nuclease activity as it is an interaction likely outside of an appropriate editing complex/substrate. Presently any potential physiological role for the DYW-deaminase linked nuclease activity in vivo remains speculative.

## Conclusions

This study supports previous experiments that have demonstrated the DYW-deaminase domain can be linked to ribonuclease activity in vitro*.* Ribonuclease activity was extended to include DYW-deaminases known to be RNA editing enzymes. This activity was greatly reduced in the presence of equimolar ratios of editing factors that are known to associate in native editing complexes. Thus, robust DYW-deaminase associated ribonuclease activity could only be observed in isolation from RIP proteins or r*At*CRR4 with cognate cis-elements in vitro, limiting the context of possible physiological functions in vivo.

## Methods

### Expression of recombinant proteins

Recombinant proteins *At*DYW1, *At*RIP2, *At*RIP9, *Zm*RIP9, *At*ORRM1, *At*CRR4 and *At*OZ1 were expressed from pET21a plasmids encoding each respective amino acid sequence downstream of N-terminal Hex-His tag. Plasmids used to express r*At*DYW1 were used from a prior study^4^. Plasmids encoding non-DYW editing factors were constructed through traditional restriction-based cloning. Phusion DNA polymerase (ThermoScientific) and oligonucleotide primers (IDT) with the sequences *At*RIP2_ForEcoRI: CAAGGAATTCATGGCTTTGCCTTTGTCTG, *At*RIP 2_RevPstI: GCAACTGCAGTCATCTTGTGTTTTCTCTGCGG, *At*RIP9_ForBamHI: GCAAGGATCCATGGCTTCCTTCACACAAC, *At*RIP9_RevHinDIII: CACAAAGCTTTTAAGAGGAATCAGAGGCTGC, *Zm*RIP9_ForBamHI: GCATGGATCCGCCGCCGCCTTCCCTGC, *Zm*RIP9_RevSalI: GCATGTCGACTCACGAAGACGCGGACTCGG, *At*ORRM1_ForBamHI: GCATGGATCCTCTTCTGCAATTTCCGCACC, *At*ORMM1_RevXhoI: GCATCTCGAGCTAGAGCCCGAAACTTGGTTG, *At*CRR4ForEcoRI: GCATGAATTCGCTTTTGCCTCTTCTCGAC, *At*CRR4RevHinDIII: GCATAAGCTTCTACAATGTACTGGAAACTTCAATGC, *At*OZ1_BamHIF: GCATGGATCCATGAACAACTCCACCAGACTC, and AtOZ1_SallR: GCATGTCGACTCATTTATCTCCTTTACCAGTGGG were used to create amplicons containing the genes for each editing factor using cDNA as template. All amplicons were cut using the appropriate restriction enzymes and directly cloned into pET21a, except in the case of *At*RIP2 which was initially cloned into pBluescriptII and then subcloned into pET21a.

Plasmids were transferred to *E. coli* Rosetta2 DE3 pLys hosts. Each strain was grown in LB broth at 37 °C to an OD600 of 0.6 and cooled by transfer to an 18 °C incubator for 30 min. Protein expression in bacterial cultures was induced by the addition to a final concentration of 1 mM IPTG and incubated at 18 °C with shaking for 4 h. Cells were harvested by centrifugation at 5000xg for 20 min and stored at − 80 °C.

### Protein purification

Cell pellets containing r*At*RIP2, r*At*RIP9, r*Zm*RIP9 and r*At*OZ1 were resuspended in a chilled lysis buffer with 50 mM HEPES pH 7.0, 250 mM NaCl, 10% glycerol, 0.01% (w/v) CHAPS, and 10 mM imidazole. Similarly, cell pellets containing r*At*DYW1 and *At*ORRM1 were resuspended in a chilled lysis buffer with 20 mM tris–HCl pH 7.3, 150 mM NaCl, 10% glycerol, 0.01% (w/v) CHAPS, and 10 mM imidazole. Cell pellets for rAtCRR4 were resuspended in 50 mM tris–HCl pH 7.3 @ RT, 250 mM NaCl, 1% glycerol, 0.01% (w/v) CHAPS, and 10 mM imidazole. PMSF suspended in 2-proponol was added to a final concentration of 1 mM before cell lysis. Cell suspensions were sonicated 6 times at 80% of the maximum amplitude for 20 s, each with 1 min of rest in between bursts. Insoluble cellular debris was removed through pelleting in the centrifuge at 12,000×*g* for 30 min. The N-terminal his-tagged proteins r*At*DYW1, r*At*RIP2, r*At*RIP9, *Zm*RIP9, r*At*ORRM1, r*At*CRR4, and r*At*OZ1 were purified using IMAC with His-PURE NiNTA resin. Proteins were assessed for purity using Coomassie stained SDS-PAGE. Size exclusion chromatography was performed with a Superdex S200 10/300 GL column (GE Life Sciences). The column was equilibrated and *At*CRR4, *At*ORRM1, *At*RIP2m *Zm*RIP9 proteins were purified using a running buffer composed of 20 mM tris–HCl pH 7.3, 150 mM NaCl, 10% glycerol at a flow rate of 0.25 mL/min.

### Immunoblotting

The samples were mixed with TruPAGE LDS Sample Buffer (Sigma-Aldrich) and electrophoresed in a 4–20% TruPAGE SDS PAGE Gel (Sigma-Aldrich). Proteins were transferred to 0.45 µm PVDF membranes by electroblotting overnight. After the transfer was completed, the membranes were incubated in 1X TBST with 5% (w/v) nonfat dry milk for an hour at room temp. Primary antibody dilutions were made in 1xTBST containing 5% milk as follows: anti-RIP9^24^ 1:5000, anti-6× His HRP Conjugate (Invitrogen) 1:2500, anti-MBP (Invitrogen) 1:2500. Membranes were incubated with each respective antibody dilution on a plate shaker at 4 °C overnight, followed by three, 10-min washes with 1× TBST at room temperature. Membranes were incubated with Peroxidase Conjugated Goat anti-Rabbit (H + L) antibody (Invitrogen) for one hour at room temperature followed by three additional 10-min washes with 1X TBST. The ProtoGlow ECL Detection Kit (National Diagnostics) and an Azure c400 gel imager (Azure Biosystems Inc.) was used to visualize the blots.

### RNA nuclease assay

Recombinant *At*DYW1 was added to a final concentration of 3.4 μM with a 5′ Cy-5 fluorescently labeled RNA at a final concentration of 15 nM under final reaction conditions of 13 mM Tris–HCl pH 7.9, 22.5 mM NaCl, 30 mM KCl, 6 mM MgCl_2_, 2 mM DTT, 10 U Ribolock RNase Inhibitor I, 37.5 mM imidazole, 25 mM EDTA, 1.5% w/v glycerol. Reactions were incubated at 28 °C for 1 h and stopped at timepoints by transfer to a − 80 °C freezer. Reaction aliquots (10 μL) were diluted with 5 µL of 3× Stop Buffer (95% (v/v) formamide, 20 mM EDTA, 0.05% (w/v) bromophenol blue, and 0.05% SDS) and loaded onto 20% denaturing polyacrylamide gel containing 6 M urea. Gels were imaged using an Azure c400 gel imager (Azure Biosystems) and band intensity calculated using ImageJ^30^.

### RNA substrate preparation for RNA editing analysis

Editing reactions were performed under standard conditions previously developed for plant organelle C-to-U RNA editing^31^. The RNA editing substrate for r*At*DYW1 contained the *ndhD* C2 editing site and sequences − 100 nt upstream/+ 5 nt downstream of the editing sites. Adapter SK and KS sequences were then added to the ends to ensure specific amplification of the RNA substrate. A PCR amplicon template to make the substrate was constructed by PCR amplification with primers AtndhDC2_SK_FOR: CGCTCTAGAACTAGTGGATCTTATTGACAAGTACTCGTACTC and AtndhDC2_KS_Rev: TCGAGGTCGACGGTATCCATTCGTGGTAAAGACAAGATAC using *Arabidopsis thaliana* gDNA as template. A second round of PCR added a 5′ T7 promoter sequence used to create the substrate using the TranscriptAid T7 High Yield Transcription Kit (ThermoScientific). The transcription product was treated with DNase I (ThermoScientific) to remove DNA while the remaining RNA was purified with an RNA Clean and Concentrator Kit (Zymo Research). Editing assays were performed in reaction mixtures (12.5 μL) that contained 30 mM HEPES–KOH (pH 7.7), 10 mM tris–HCl, 3 mM magnesium acetate, 75 mM NaCl, 45 mM potassium acetate, 30 mM ammonium acetate, 1 mM ATP, 5 mM dithiothreitol, 1% Polyethylene 16 Glycol 6000, 10% glycerol, 0.005% (w/v) CHAPS, 20 mM imidazole, 30 U of RiboLockTM RNase inhibitor (ThermoScientific), 1× Complete proteinase inhibitor mixture (Boehringer Mannheim), 1 fmol of mRNA substrate, and 6.25 μl purified protein cocktail. Reactions with 6 RNA editing factors included final concentrations of 2 μM r*At*DYW1, 0.3 μM r*At*CRR4, 1 μM r*At*RIP2, 2 μM r*At*RIP9, 2.7 μM r*At*OZ1, and 0.7 μM r*At*ORRM. Reactions without r*At*DYW1 but containing other editing factors included final concentrations of 0.4 μM r*At*CRR4, 1.2 μM r*At*RIP9, 3.2 μM r*At*OZ1, and 0.8 μM r*At*ORRM. Editing was assayed using an adapted quantitative poisoned primer extension assay^32^ modified by the use of fluorescent instead of ^32^P radiolabeled oligonucleotides.

## Supplementary Information

Supplementary Figures.

## Data Availability

The datasets used and/or analyzed during the current study available from the corresponding author on reasonable request.

## Abbreviations

PPR: Pentatricopeptide repeat
RIP/MORF: RNA editing factor interacting proteins/multiple organellar RNA editing factors
ORRM: Organelle RNA recognition motif
OZ: Organellar zinc-finger
CRR: Chlororespiratory reduction
THU: Tetrahydrouridine
OTP85: Organelle transcript processing 85
EDTA: Ethylenediaminetetraacetic acid
PMSF: Phenylmethanesulfonyl fluoride
ATP: Adenosine 5′-triphosphate
CMP: Cytidine 5′-monophosphate
PCR: Polymerase chain reaction
*ndhD*: NADH dehydrogenase subunit 4
*rps14*: Ribosomal protein S14
*ndhB*: NADH dehydrogenase subunit 2
*rps7*: Ribosomal protein S7
Ni-NTA: Nickel-nitrilotriacetic acid
IMAC: Immobilized metal affinity chromatography
CHAPS: 3-[(3-Cholamidopropyl)dimethylammonio]-1-propanesulfonate
