# Supplementary material for: A ribonuclease activity linked to DYW1 in vitro is inhibited by RIP/MORF proteins
Source: Sci Rep. 2023 Jul 3;13:10723. doi: 10.1038/s41598-023-36969-6 (PMC10318007; doi:10.1038/s41598-023-36969-6)
Supplement: Supplementary file 1 — Supplementary Figures. [file 41598_2023_36969_MOESM1_ESM.pdf]

# Supplementary Information

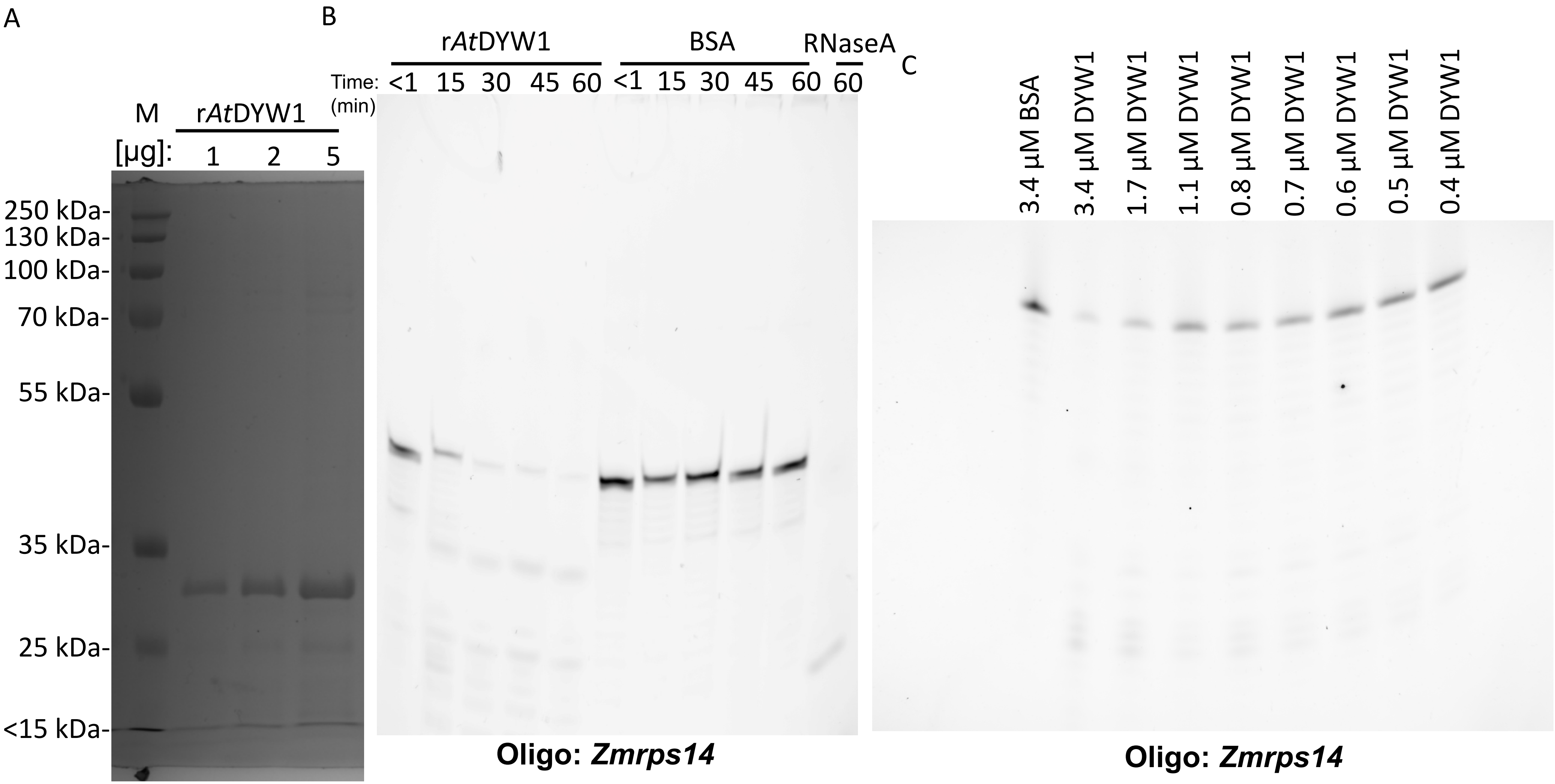


Fig. S1. Raw images used from cropped images in Fig. 1A (**A**) where 1, 2, and 5 µg of r*At*DYW1 was added, Fig. 1B (**B**), and Fig. 1D (**C**).

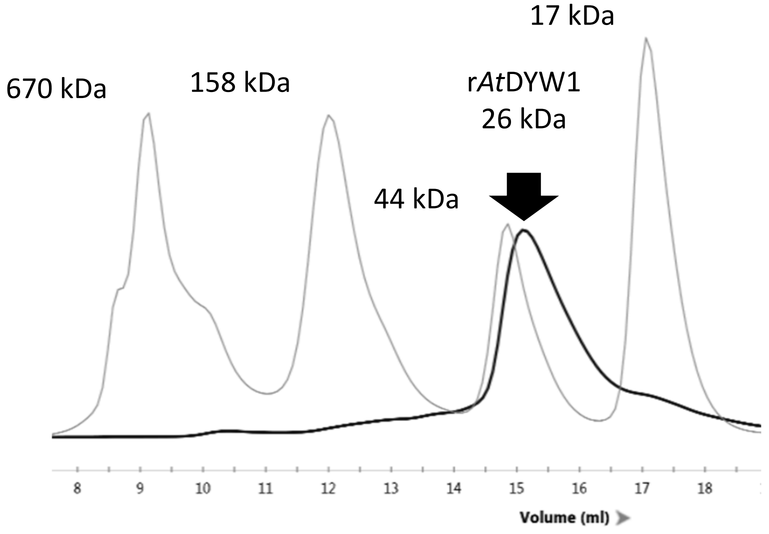


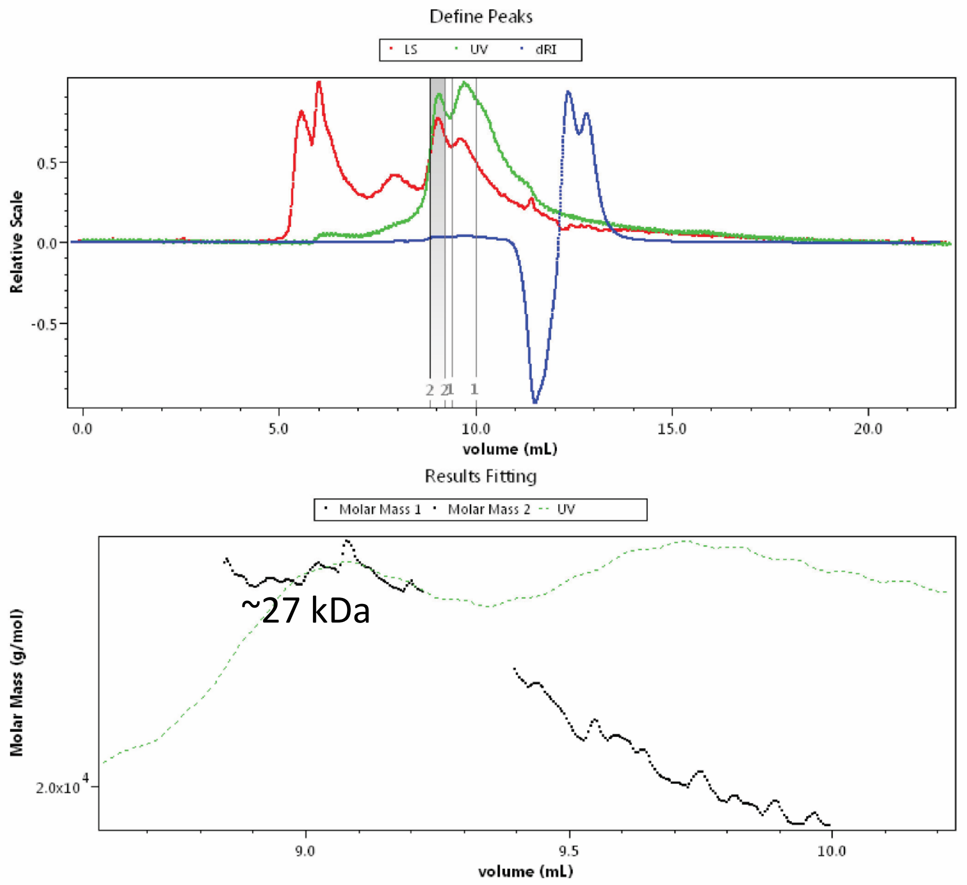


Fig. S2. Recombinant *At*DYW1 could be resolved as a monomer on Superdex S200 media size exclusion chromatography (at left) and its molecular weight could be estimated using a multiangle light scattering (at right).

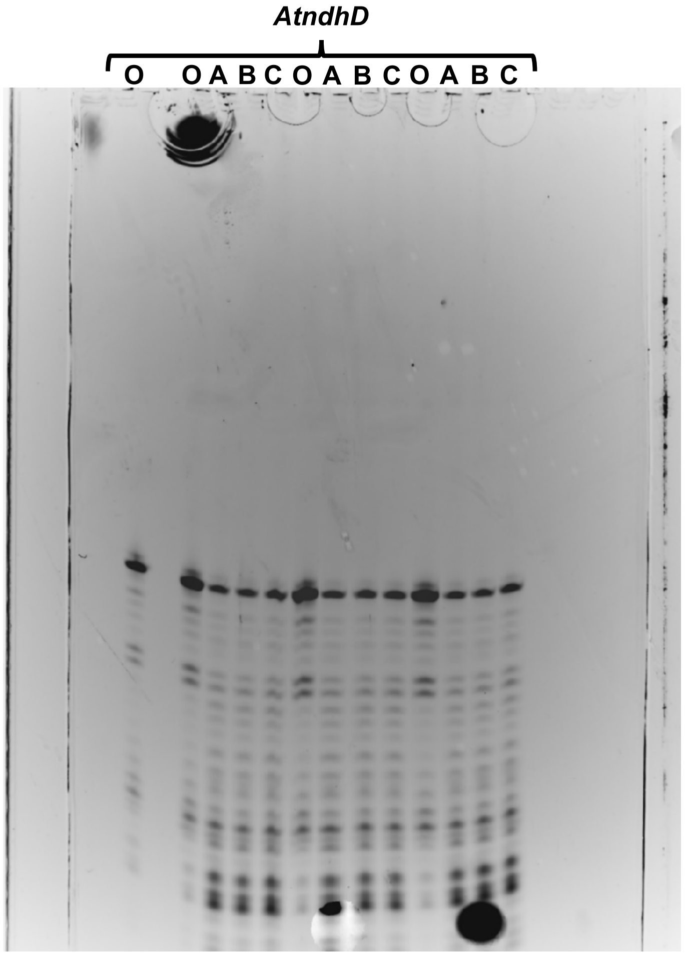


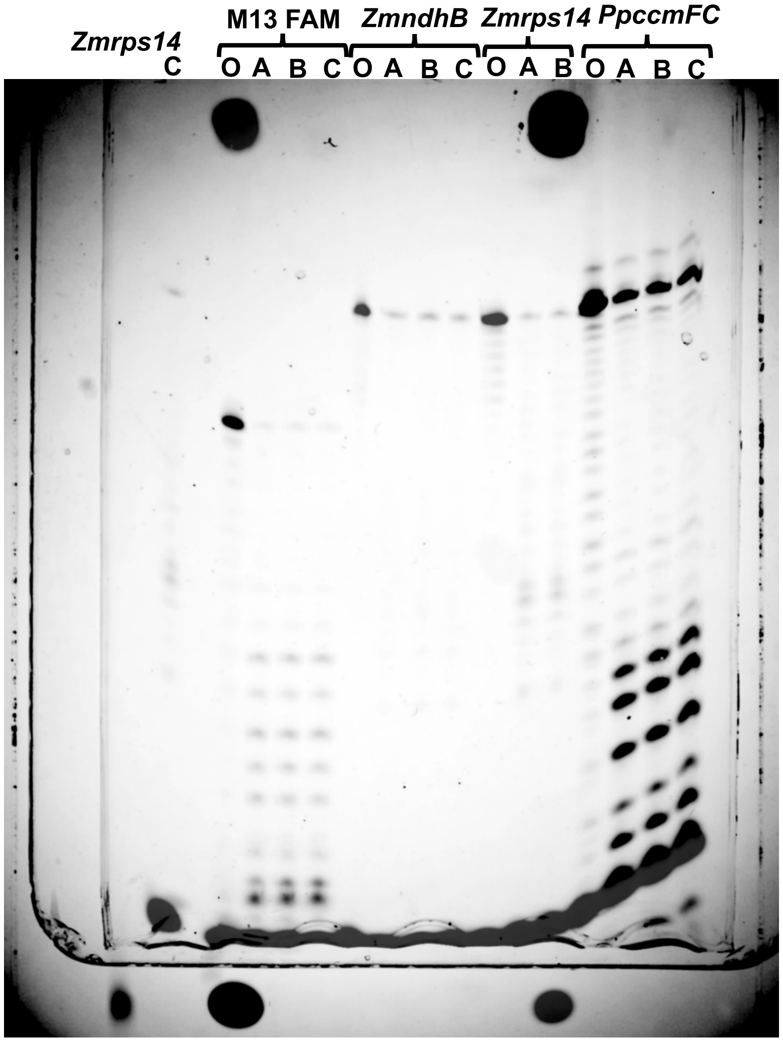


Fig. S3. Raw images used from cropped images in Fig. 2. The oligonucleotide lane is labeled (O) with replicate reaction lanes labeled A, B, and C. Oligo names are written above replicate lanes.

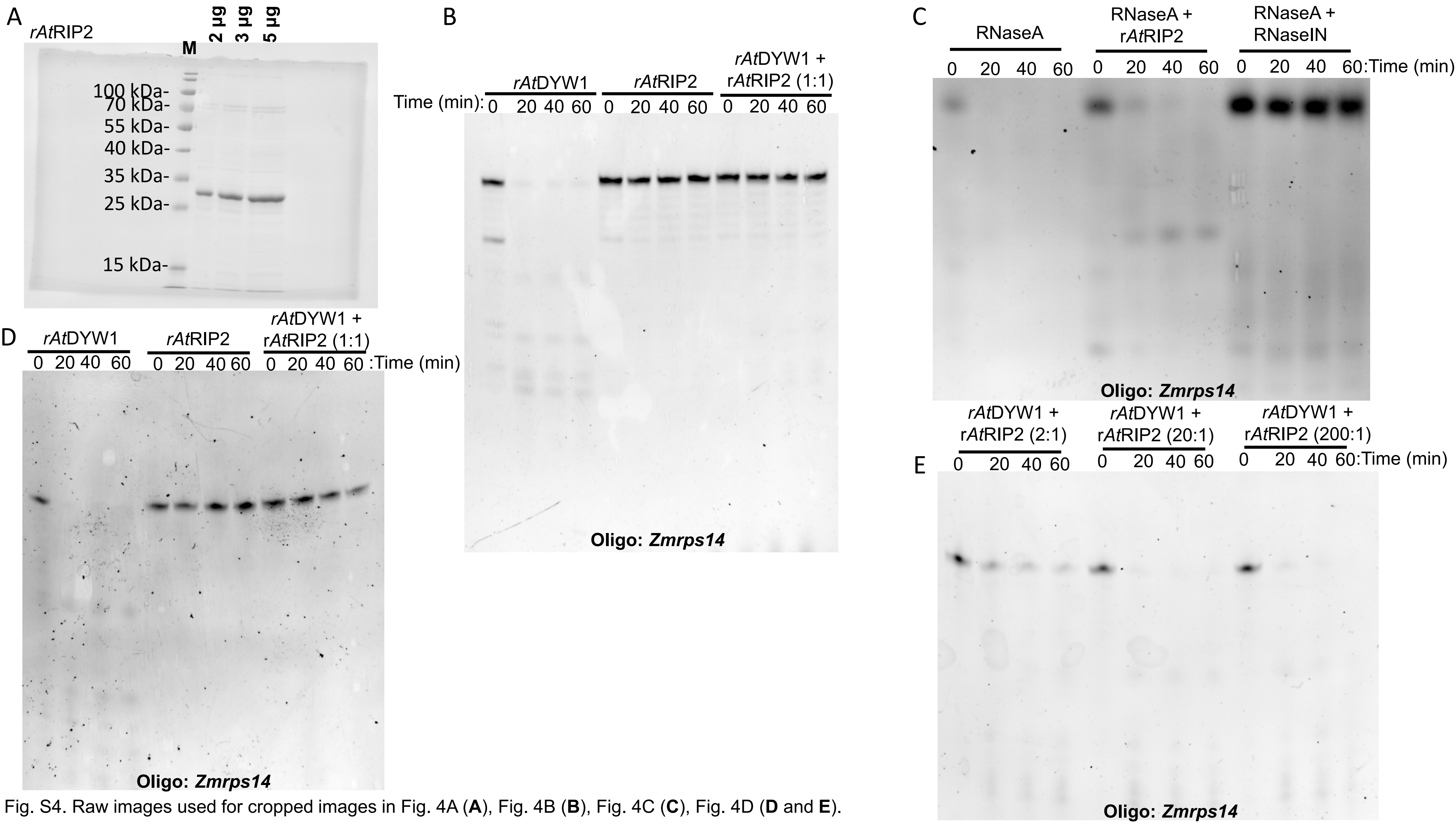


Fig. S4. Raw images used for cropped images in Fig. 4A (**A**), Fig. 4B (**B**), Fig. 4C (**C**), Fig. 4D (**D** and **E**).

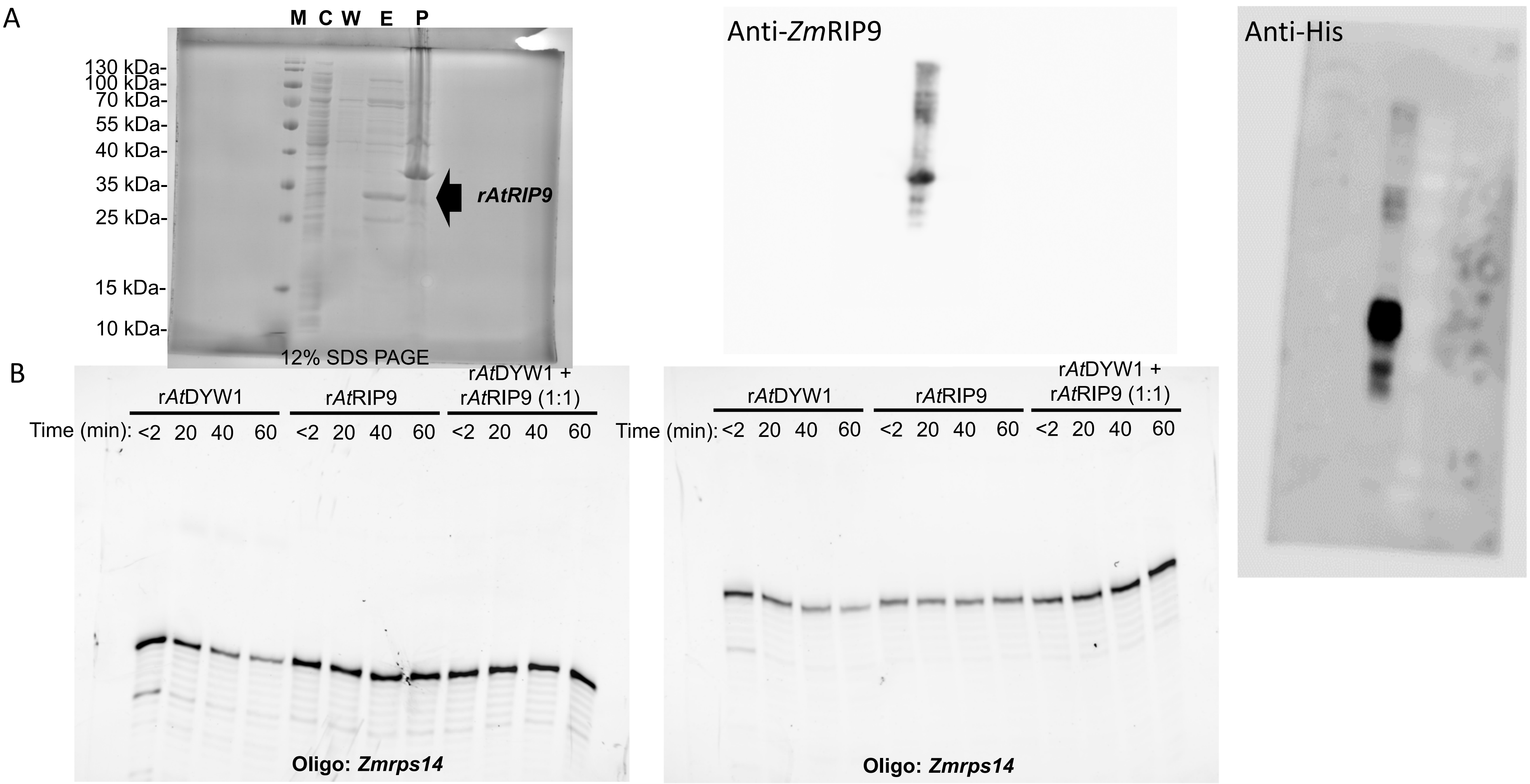


Fig. S5. Recombinant r*At*RIP9 strongly inhibits r*At*DYW1 activity. **A** Crude fractions of recombinant *At*RIP9 could be purified by a single round of IMAC but fractions contained degradation products and contaminating bands. At left, an image of a Coomassie-stained SDS-PAGE indicates different sized protein products in purified fractions. In the middle, an immunoblot of r*At*RIP9 enriched fractions using Anti-RIP9 polyclonal antibodies developed against a *Zm*RIP9 amino acid sequences from a previous study [24]. At right, an immunoblot on the same fraction using a commercially derived monoclonal Anti-His antibody. Molarity for r*At*RIP9 in fractions was estimated based on the molecular weight of full length r*At*Rip9 and total protein concentration. **B** Images of labeled *Zmrps14* RNA oligonucleotides separated on 6M urea 20% PAGE from duplicate reactions with r*At*DYW1, r*At*RIP9, and an equimolar mixture of r*At*DYW1 and r*At*RIP9.

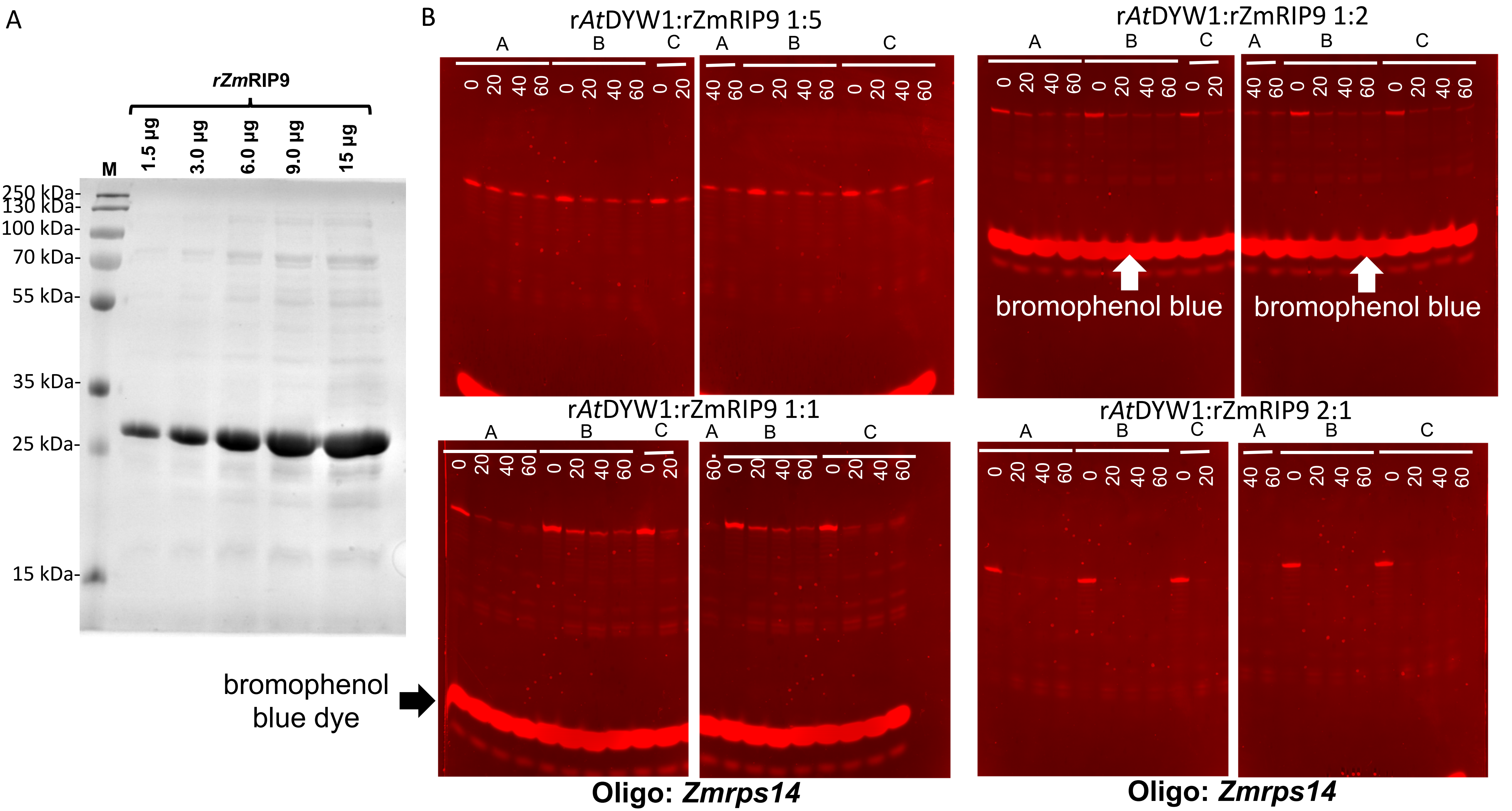


Fig. S6. Raw images used in Fig. 5a (**A**), and Fig. 5b (**B**). Two images of the same gel are shown for each panel in **B** for triplicate reactions A, B, and C. For some gel images heavy bands at the bottom of gels can be seen due to the bromophenol blue dye used in the sample buffer. The red channel is shown in this example before conversion to grayscale.

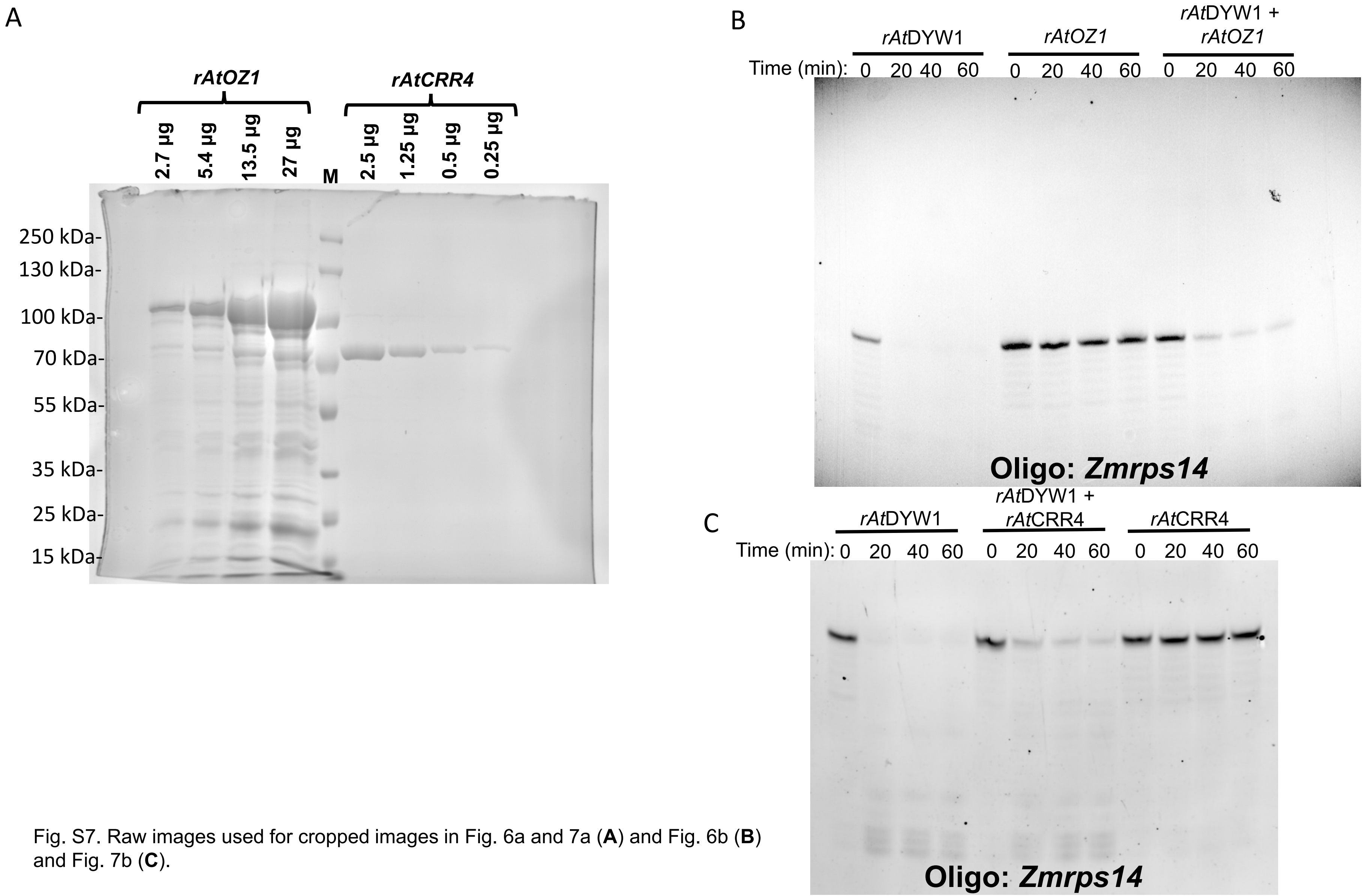


Fig. S7. Raw images used for cropped images in Fig. 6a and 7a (**A**) and Fig. 6b (**B**) and Fig. 7b (**C**).

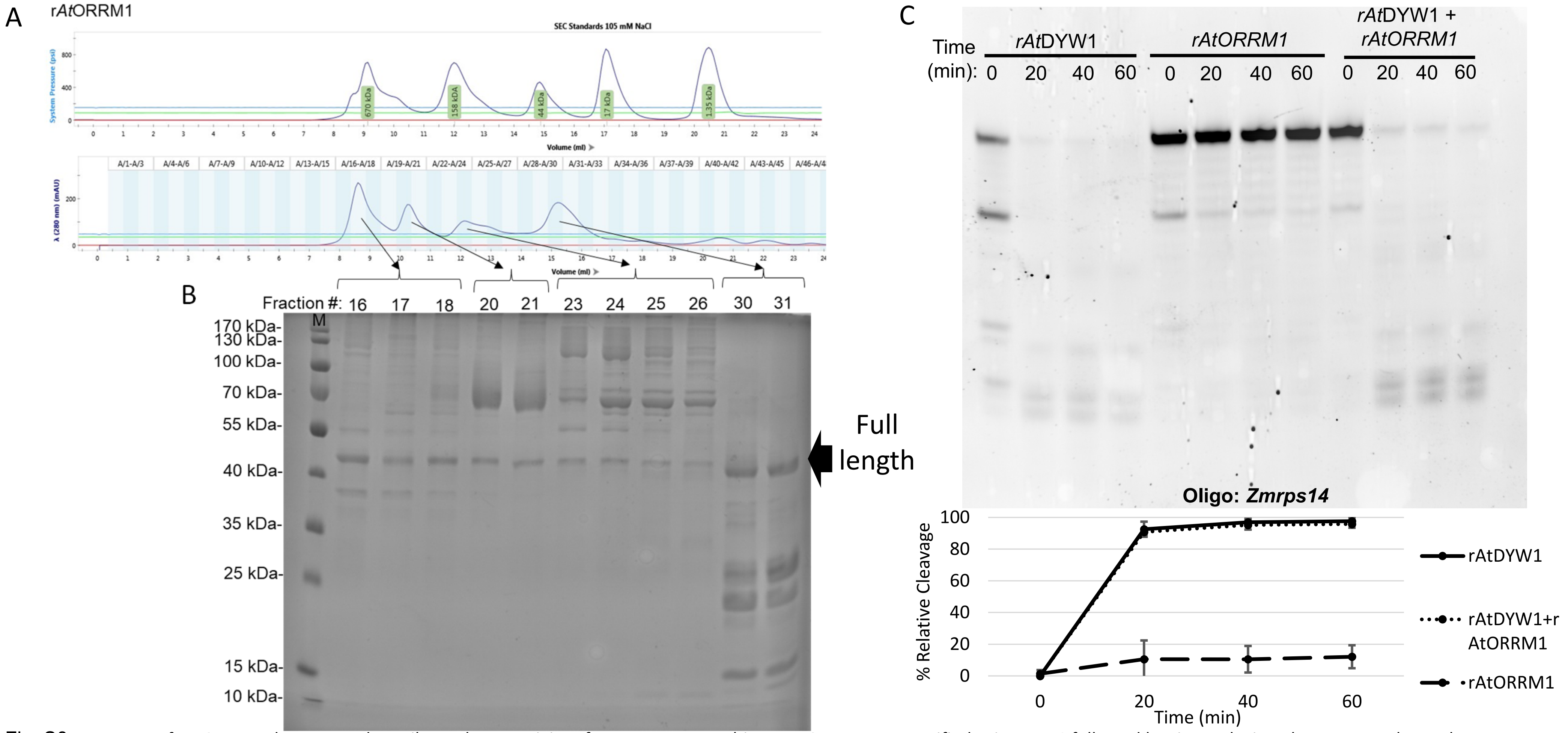


Fig. S8. Presence of r*At*ORRM1 does not reduce ribonuclease activity of r*At*DYW1. Recombinant *At*ORRM1 was purified using IMAC followed by size exclusion chromatography. **A** The trace from the gel filtration experiment at 280 nm is shown for Gel filtration standards (at top) and the enriched r*At*ORRM1 fraction (at bottom). **B** Fractions with a peak absorbance that correlated with estimates for monomeric r*At*ORRM1 were separated by SDS-PAGE. An image of the Coomassie-stained gel represents the purify level of fractions after two rounds of chromatography and displays several likely degradation products. A band that correlates with the estimated size of full length r*At*ORMM1 is marked by the arrow. The molarity of r*At*ORRM1 was estimated based on the full-length molecular weight and the total protein concentration of the fraction. **C** Ribonuclease activity was assayed in triplicate reactions where RNA *Zmrps14* oligonucleotides were mixed r*At*DYW1, r*At*ORRM1, and r*At*DYW1 + r*At*ORRM1 at roughly equimolar concentrations. A representative 6M urea gel image is shown (at top), and the data from all reactions is displayed in the X-Y scatterplot (at bottom). Error bars represent one standard deviation from the mean.

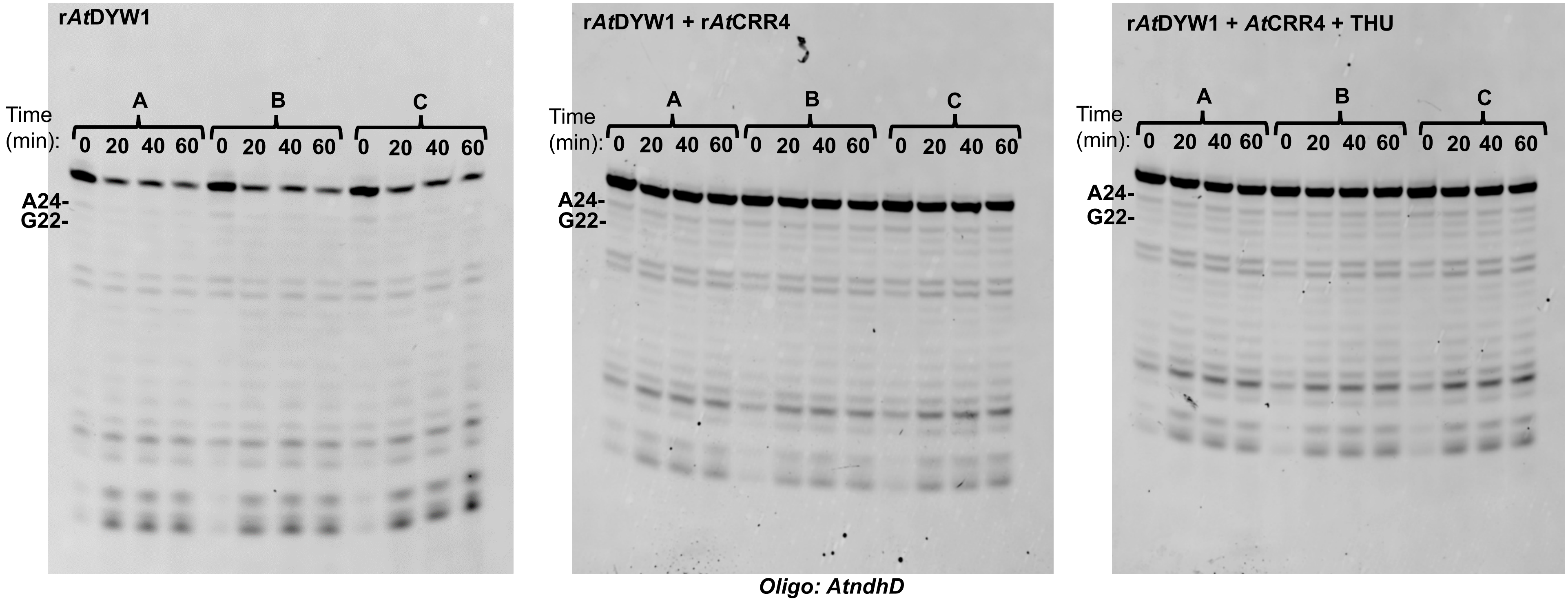


Fig. S9. An expanded image from Figure 7 showing the pattern of RNA species generated in r*At*DYW1 reactions. Oligonucleotides with *AtndhD* sequences were added to r*At*DYW1 alone (top), r*At*DYW1 with equimolar r*At*CRR4 (middle), and r*At*DYW1 with equimolar r*At*CRR4 in the presence of THU. Reactions were run in triplicate and lanes are labeled for each reaction with A, B, C. RNA species were separated on 20% PAGE with 6M urea.

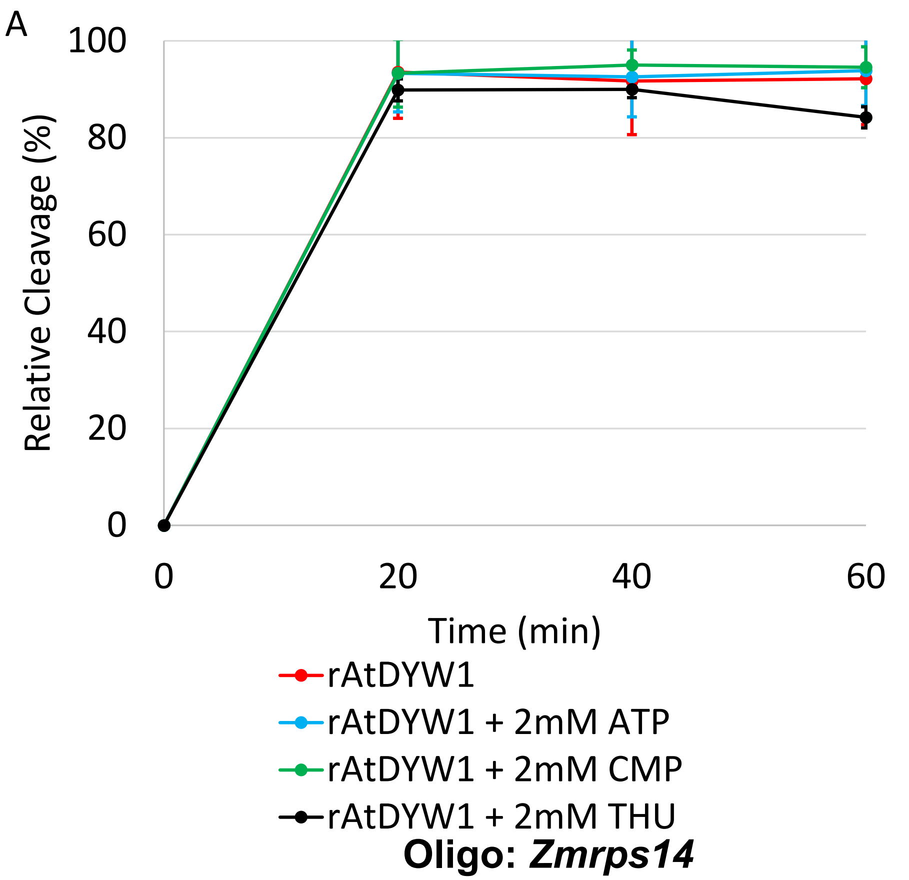


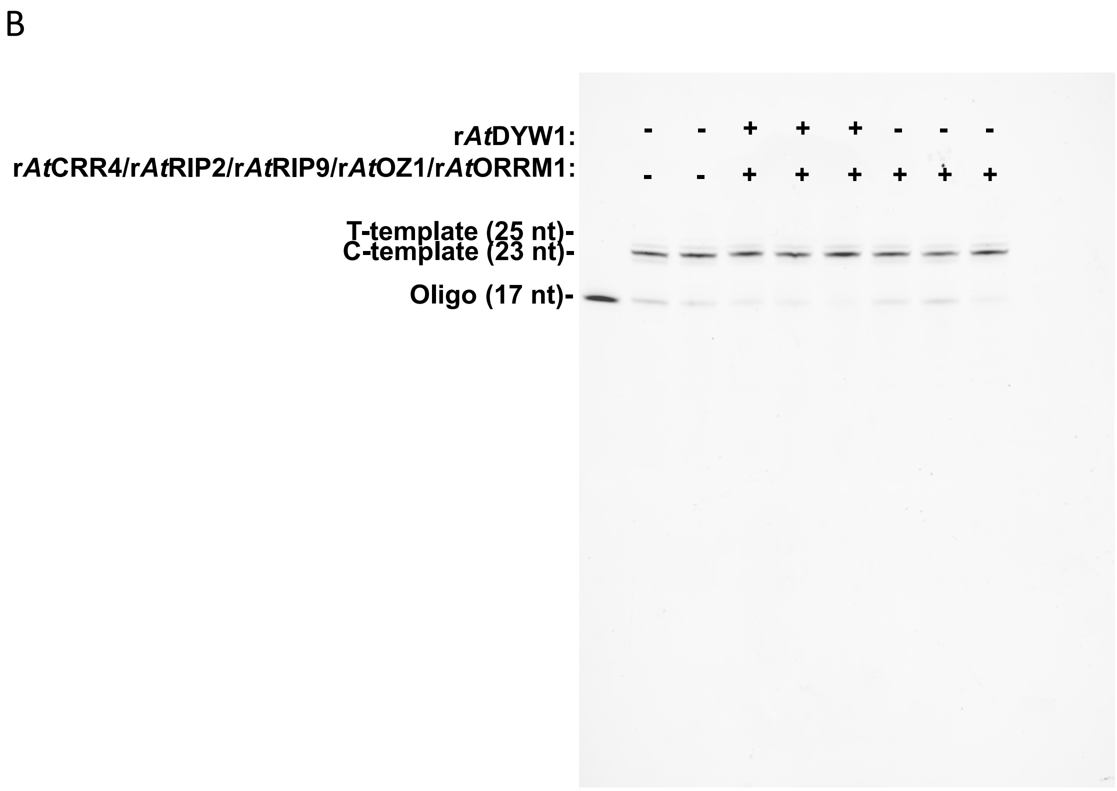


Fig. S10. **A** An X-Y scatterplot represents % relative cleavage of RNA oligonucleotides with *Zmrps14* sequences in triplicate reactions over 60 min in the presence of 2 mM CMP, ATP, and THU. Error bars represent 1 standard deviation from the mean from triplicate reactions. **B** Editing factors were mixed in equimolar ratios with *ndhD* derived substrates and the RNAs were then used as template for RT-PCR. Amplicons were purified and poisoned primer reactions were used to generate template specific products that contained a 5' tetrachlorofluorescein probe. An image of the primer extension products is shown with lanes from left to right resulting from reactions with no r*At*DYW1 or accessory factors, r*At*DYW1 with accessory factors r*At*CRR4/r*At*RIP2/r*At*RIP9/r*At*OZ1/r*At*ORRM1, and accessory factors lacking the r*At*DYW1 enzyme. The location of the expected product from a C containing template that would result from unedited RNA are labeled C-template and the proximal location of a T containing template product that would result from edited RNA is labeled T-template. Editing factors were added to putative *AtndhD* substrates in triplicate reactions.
